# Maternal and Neonatal Outcome of Surviving Twin after Single Fetal Demise at 17 Weeks: A Case Report

**DOI:** 10.31729/jnma.9204

**Published:** 2025-08-31

**Authors:** Puja Thakur, Nischal Joshi, Preeti Kumari Sah, Manikant Thakur, Rashmi Kumari Mandal, Jagat Bahadur Thapa, Shweta Jaiswal

**Affiliations:** 1 Patan Academy of Health Sciences, Lagankhel, Lalitpur; 2 Department Of Obstetrics and Gynecology, Patan Academy of Health Sciences

**Keywords:** *intrauterine fetal demise*, *monochorionic diamniotic twins*, *single fetal demise*

## Abstract

Single fetal demise in a monochorionic diamniotic twin pregnancy poses significant risks to both mother and the surviving twin. We report a monochorionic diamniotic twin pregnancy where one fetus demised at 17 weeks, and pregnancy was prolonged by 10 weeks with expectant management. At 28+3 weeks, an emergency cesarean section was performed for preterm premature rupture of membranes. The mother had no major complications. The surviving twin weighed 1350 grams at birth and required neonatal intensive care unit care for respiratory distress and sepsis but showed significant improvement and was discharged after two months. This case is unique due to 10-week prolongation after single intrauterine fetal death, especially with favorable maternal and neonatal outcome.

## INTRODUCTION

The incidences of multiple gestation has risen 3-4% worldwide.^1^ Single fetal demise in twin pregnancies occurs in 0.5-6.8% of cases, with greater risks in shared placenta.^2^ MCDA (monochorionic diamniotic) pregnancies carry risk like preterm birth, restricted growth, severe neurological impairment and intrauterine fetal demise (IUFD) of surviving twin and maternal complications like antepartum bleeding, septic shock, DIC, heart failure and maternal death.^3-5^ Expectant management beyond a few weeks is uncommon and continuation of such pregnancies requires careful monitoring and management to optimize outcomes and is usually under 5 weeks.^4^

This case is reported for its rarity, a 10-week prolongation after single IUFD with favorable outcomes despite multiple maternal risk factors.

## CASE REPORT

A 36-year-old woman, G4P1L1A2, at 23+3 weeks with MCDA twins, chronic hypertension, prior cesarean delivery, obesity, and history of infertility presented with ultrasound-confirmed demise of one twin. The surviving twin appeared normal. She was taking amlodipine and aspirin for her comorbidities.

On admission, she was alert and oriented with stable vitals. No pallor, icterus, or edema was noted. Fundal height corresponded to 26 weeks; fetal parts were palpable, and fetal heart sounds were heard for one twin at 150 bpm.

Anomaly scan at 18 weeks had shown one live fetus (18+4 weeks) and another (17+4 weeks) without cardiac activity. Repeat scan at 23 weeks, done at our center, confirmed these findings without evidence of hydrops fetalis, twin-to-twin transfusion or reversed arterial perfusion.

The patient and her family were counseled about risks, including severe neurological impairment, double IUFD, and maternal complications. Despite advice for termination, they opted for continuation of pregnancy. She was admitted, monitored with serial ultrasound scans, and given antenatal corticosteroids to enhance fetal lung maturity. Multidisciplinary reviews were done for her chronic health conditions.

At 28+3 weeks due to preterm premature rupture of membranes, an emergency cesarean section was performed. A male child weighing 1350 grams was delivered with APGAR scores of 7/10 and 8/10. The other twin was anomalous and showed no signs of living activity. The sex of the demised twin could not be determined.

The mother recovered uneventfully and was discharged on day three. The neonate required NICU care for respiratory distress (treated with surfactant), hyperkalemia, septic shock, AKI, left lung collapse, apnea of prematurity, pneumonia and retinopathy of prematurity of stage II. During hospital course, the child received supportive therapy along with antibiotics, He improved and was discharged after two months weighing 2180 grams in a stable condition with no active complication.

**Figure 1:**
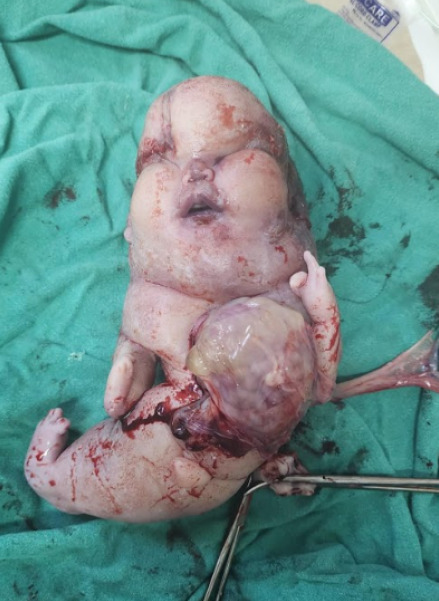
Gross appearance of the deceased co-twin delivered in MCDA pregnancy.

## DISCUSSION

Single fetal demise in twin pregnancies, particularly in monochorionic diamniotic (MCDA) twins, presents a unique and challenging clinical situation. While single fetal demise before 17 weeks is usually uneventful, the death of a twin in late second or third trimester is a rare obstetric complication.^6^ The risk of morbidity and mortality in the surviving twin is substantially higher in monochorionic pregnancies compared to dichorionic ones, due to the shared placental circulation. Study has demonstrated that the surviving twin in monochorionic pregnancies has a 12% risk of death, compared to a 4% risk in dichorionic twins, highlighting the delicate nature of such cases.^7^ The continuation of pregnancy beyond 5 weeks is more risky and less reported.^4^ Further, single intrauterine fetal demise before 22 weeks of gestation has been linked to extremely unfavorable outcome in the surviving co-twin with double twin demise the most likely outcome.^8^

A retrospective study in 2024 reported that single IUFD is associated with higher risks of preterm labor, preeclampsia, and adverse neonatal outcomes, findings consistent with our case.^9^

In the presented case, the patient opted for expectant management despite persistent counseling regarding the potential risks of neurological impairment in the surviving twin and severe maternal complications. This decision highlights the importance of patient autonomy and the need for clear and compassionate communication in high-risk obstetric care.

Expectant management was done primarily to prolong gestation, to allow further fetal maturation, especially pulmonary development. Serial ultrasonography monitoring, with careful maternal observation, and administration of antenatal corticosteroids were done for it. Despite the pre-term premature rupture of membranes (PPROM) at 28+^3^ weeks requiring emergency cesarean delivery, the surviving twin had reasonable APGAR scores and birth weight consistent with gestational age, suggesting effective intrauterine management. This is consistent with preterm delivery being the most common complication associated with single intrauterine fetal demise.^5^

In contrast to our case, previous reports have shown neonatal mortality rates as high as 29% and neurodevelopmental disorders in 7-12% of surviving co-twins. In our case, the neonate had a favorable outcome with reassuring APGAR scores and no immediate neurological deficits, likely due to close fetal monitoring and timely delivery.^2,10^

Previous studies suggest that neurodevelopmental morbidity remains the most frequent postnatal complication, with the role of chorionicity having conflicting data. Several reports indicate that conditions such as twin-to-twin transfusion syndrome or vascular anastomoses increase the risk of neurological morbidity in the surviving twin.^3,5,9,11^ In our case, serial antenatal Doppler scans revealed no evidence of such complications. This absence likely contributed to the favorable neurological status observed at birth. This outcome shows the critical role of vigilant antenatal surveillance and timely interventions in improving the prognosis.

However, these infants remain at a high risk category for later neurodevelopmental delays.^9^ Therefore, postnatal follow-ups are necessary to evaluate the developmental milestones to ensure early detection and intervention if any such deficits arise. This approach not only supports optimal long-term outcomes but also provides reassurance to families regarding ongoing care and potential monitoring needs.

In this case, the absence of maternal postoperative complications despite multiple risk factors, highlights the effectiveness of careful monitoring and proactive management strategies.

This case report supports that, with appropriate multidisciplinary management and close surveillance, favorable maternal and neonatal outcomes can be achieved even in such highrisk scenarios. Further research and accumulation of similar case reports could help establish clearer guidelines for the management of such rare but critical conditions.

## Consents:

JNMA Case Report Consent Form was signed by the patient and the original article is attached with the patient’s chart.
